# ‘Acute Heart Failure’: Should We Abandon the Term Altogether?

**DOI:** 10.1007/s11897-022-00576-9

**Published:** 2022-09-27

**Authors:** Sam Straw, Andreas Napp, Klaus K. Witte

**Affiliations:** 1 Leeds Institute of Cardiovascular and Metabolic Medicine, University of Leeds, Leeds, UK; 2 Department of Internal Medicine I, University Clinic, RWTH Aachen University, Aachen, Germany

**Keywords:** Acute heart failure, Decompensation, Guideline-directed medical therapy, Diuretics

## Abstract

**Purpose of Review:**

The distinction between ‘acute’ and ‘chronic’ heart failure persists. Our review aims to explore whether reclassifying heart failure decompensation more accurately as an event within the natural history of chronic heart failure has the potential to improve outcomes.

**Recent Findings:**

Although hospitalisation for worsening heart failure confers a poor prognosis, much of this reflects chronic disease severity. Most patients survive hospitalisation with most deaths occurring in the post-discharge ‘vulnerable phase’. Current evidence supports four classes of medications proven to reduce cardiovascular mortality for those who have heart failure with a reduced ejection fraction, with recent trials suggesting worsening heart failure events are opportunities to optimise these therapies.

**Summary:**

Abandoning the term ‘acute heart failure’ has the potential to give greater priority to initiating proven pharmacological and device therapies during decompensation episodes, in order to improve outcomes for those who are at the greatest risk.

## Introduction

Acute heart failure is a misnomer. The term describes the rapid onset or worsening of symptoms, severe enough for a patient to seek urgent medical attention [1]. But what exactly makes heart failure *acute*? It cannot be the requirement for hospitalisation, since, although many patients are hospitalised, an increasing number undergo urgent evaluation and augmentation of their therapies in the community mitigating the need for admission. Is it the rapidity of deterioration? *Acute* implies a disease process occurring within minutes or hours, but in heart failure, this is seldom the case. Most patients are already known to have established chronic heart failure, and even when hospitalisation is the first time that cardiac structure and function are assessed, intravascular volume expansion and changes in left ventricular volumes are likely to have occurred over the preceding days or weeks. Furthermore, at what point does a patient with *acute* heart failure become a patient who has *chronic* heart failure? Should it be at discharge, or when the patient no longer requires intravenous diuretics, or perhaps when results of the echocardiogram are known since even in patients rendered asymptomatic, heart failure cannot be cured? [2].

Despite these contradictions, the distinction between acute and chronic heart failure persists, the consequences of which have been two-fold. Firstly, it has resulted in (unsuccessful) efforts to identify therapies specifically designed for administration during decompensations which might improve subsequent prognosis. Secondly, it has caused many to view acute heart failure as a distinct clinical entity, the implications being that hospital-based teams need not concern themselves with initiating and optimising disease modifying therapies, since this can be deferred until after discharge. This review will summarise how reclassifying acute heart failure more accurately as an event within the natural history of chronic heart failure could improve prognosis further by giving greater priority to initiating and optimising proven therapies for those at the highest risk [3].

## The Relationship Between Hospitalisation and Prognosis

The observation that worsening heart failure events are associated with an increased risk of re-hospitalisation and mortality led to the hypothesis that these events are causally linked to disease progression and outcomes. One proposed mechanism was that acute left ventricular distension during periods of decompensation could lead to myocardial injury or ischaemia. Were this the case, rapid reversal of raised filling pressures might preserve myocardial viability and improve subsequent prognosis. However, agents proven to successfully achieve rapid reductions in right atrial or pulmonary capillary wedge pressures and alleviation of dyspnoea in hospitalised patients such as ularitide [4, 5] and serelaxin [6] have not translated into reductions in cardiovascular mortality in phase III trials compared with a more simple (and considerably less expensive) diuretic approach [7, 8].

**Fig. 1:**
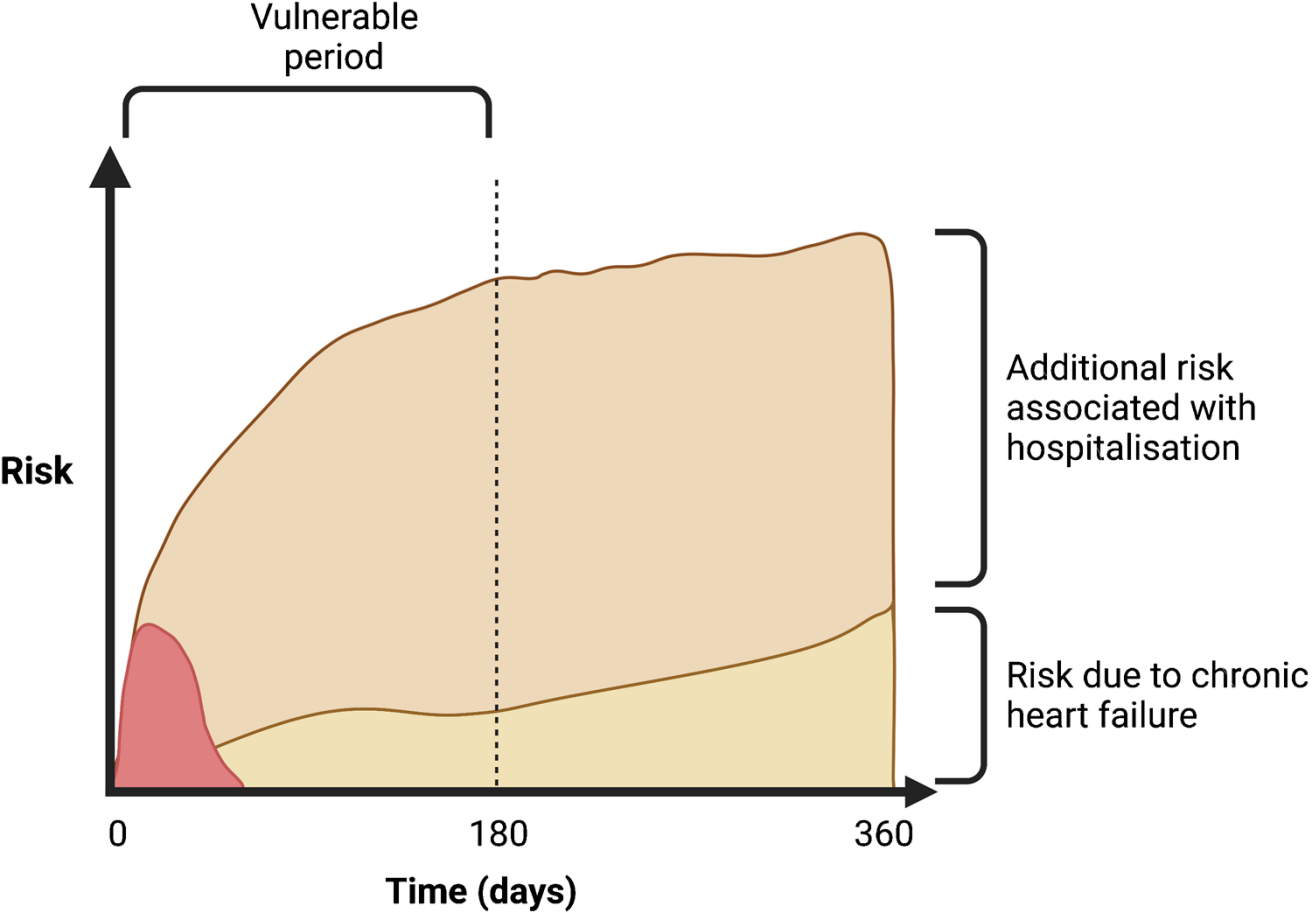
Increased risk of mortality associated with hospitalisation with worsening heart failure. Few patients die during hospitalisation (red), the majority die following discharge (brown), especially during the vulnerable phase during which the risk is many times higher compared to similar patients who are never hospitalised (yellow)

Importantly, a consistent observation is that despite the poor overall prognosis associated with worsening heart failure events, the majority of patients improve (at least initially) with standard care and most survive until discharge. In a large representative population from the USA, patients who required admission but in whom care was limited to intravenous diuretics experienced an in-hospital mortality rate of only 1.6% [9]. Even for patients who required intensification of treatment during the hospitalisation (usually an increase in the dose of loop diuretics or combination therapy with other diuretics), the pre-discharge mortality was only 12.4%. Instead, most adverse events associated with hospitalisation episodes occur within the ‘vulnerable phase’ following discharge, particularly within the first 180 days (Fig. 1) [10].

Since most patients survive until discharge, despite clear evidence that hospitalisation is a marker of adverse prognosis, these events might simply reflect (chronic) disease severity. In an analysis of patients hospitalised with *worsening* heart failure in an international cohort study, the association between hospitalisation and 180-day mortality (hazard ratio [HR] 1.35, 95% confidence interval [CI] 1.14–1.59; *p* < 0.01) was attenuated once adjusted for relevant patient characteristics measured at the time of admission (HR 1.18, 95% CI 0.99–1.40; *p* = 0.064) [11]. Intriguingly, this suggests that hospitalisation itself is not causally related to outcomes [7]. Instead, in addition to focussing on alleviating symptoms during decompensation, hospitalisation should be seen as an opportunity to optimise long-term care for those at the greatest future risk.

## How Do Patients with Worsening Heart Failure Present?

**Fig. 2:**
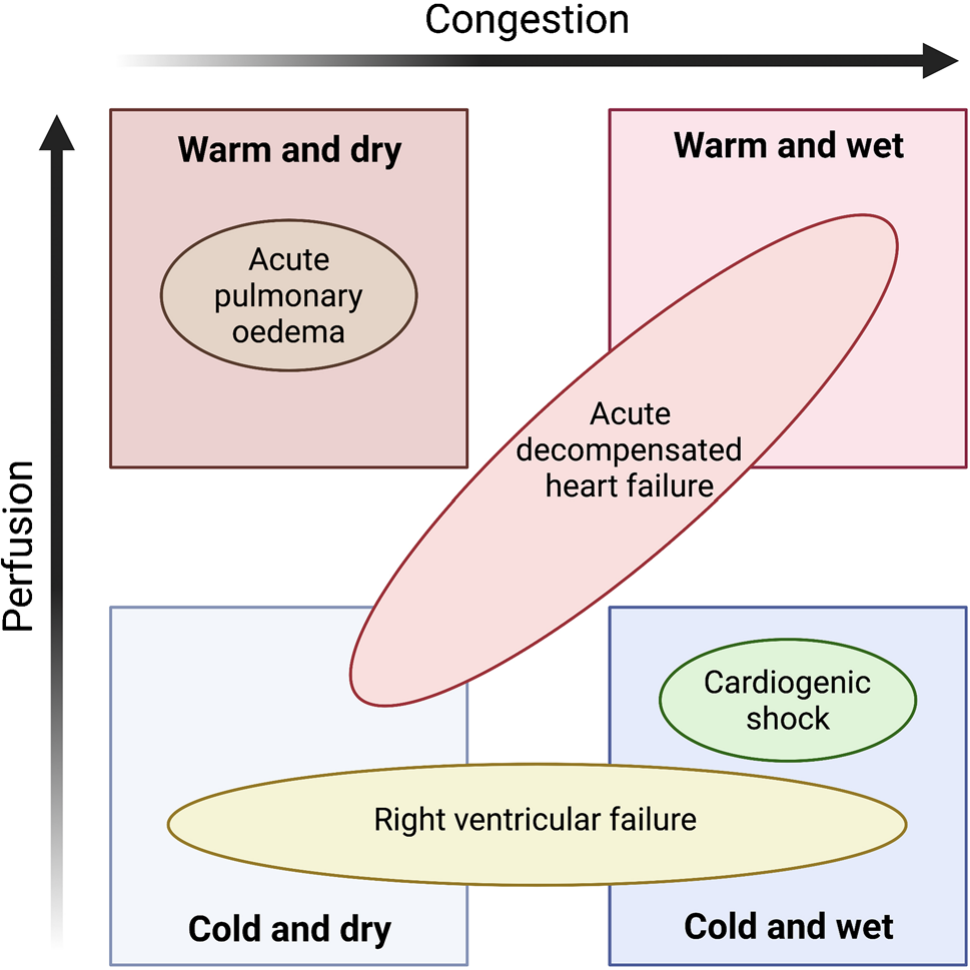
Conceptual frameworks of acute heart failure presentations. Patients may be classified based on perfusion (warm or cold) and congestion (dry or wet) or based on pathophysiological mechanisms

Patients with worsening heart failure typically have symptoms of dyspnoea and congestion. However, the clinical signs and underlying pathophysiology vary considerably. Two conceptual frameworks have been developed to classify presentations, the first of which distinguishes patients by the presence or absence of congestion (‘wet’ or ‘dry’) and peripheral hypoperfusion (‘warm’ or ‘cold’) (Fig. 2) [12]. Although this framework is simple, it provides limited insight. The vast majority of decompensated patients are ‘wet and warm’ whilst few are ‘cold and dry’ [13]. Furthermore, it is not conceivable that patients who are neither congested nor hypoperfused (‘warm and dry’) could be viewed as being decompensated. More importantly, there can be significant overlap in the clinical signs of patients presenting with distinct phenotypes who may require quite different approaches.

Hence, this framework has been superseded by criteria based on the pathophysiological mechanisms driving heart failure decompensations, which describes the four most commonly encountered clinical scenarios. Acute decompensated heart failure (ADHF) accounts for the majority of patients presenting with symptoms of worsening heart failure. Such patients have established chronic heart failure, which worsens gradually over days or weeks, most of whom are ‘wet and warm’. Acute pulmonary oedema typically presents more rapidly, is often a first presentation, and is usually not associated with peripheral oedema. Isolated right ventricular failure is another commonly encountered clinical presentation, the predominant symptom being systemic congestion, with or without hypoperfusion. Finally, cardiogenic shock accounts for a small number of patients who have a particularly poor prognosis. Cardiogenic shock is rarely simply ‘heart failure’ — more frequently, it is an acute presentation of a new event such as myocardial infarction, ventricular arrhythmia, fulfilment myocarditis, or Takotsubo cardiomyopathy. Patients presenting with a cardiogenic shock picture require specific therapies, intensification of therapies beyond intravenous diuretics, and typically have clinical signs of hypoperfusion and congestion.

## Can We Prevent Hospitalisation in Patients with Worsening Heart Failure?

Not only is hospitalisation costly [14], it worsens health-related quality of life [15], and although most patients survive hospitalisation, around a third die within 1 year of discharge [11, 16] most commonly during the ‘vulnerable phase’ [10]. Since the majority of patients admitted with worsening heart failure have established chronic heart failure, the ideal strategy for patients and healthcare systems would be to identify and optimise care for patients at risk with a view to avoiding hospitalisation altogether. One possible approach is remote monitoring either through previously implanted cardiac devices (including pacemakers, cardiac resynchronisation therapy [CRT], and implantable cardioverter defibrillators [ICDs]) or devices specifically and solely designed for monitoring physiological variables.

### Remote Monitoring Using Implantable Cardiac Devices

The largest study of remote monitoring using implantable devices — the REmote Monitoring of Heart Failure (REM-HF) trial, assessed whether information gained from a protocolised remote monitoring strategy could improve outcomes for patients with established chronic heart failure [17]. In a representative population with implanted devices, an active remote monitoring strategy generated greater clinical activity but did not improve survival or reduce hospitalisations, which were in fact more commonly observed for patients in the active arm who had persistent or permanent atrial fibrillation [18]. The Sensitivity of the InSync OptiVol feature for the prediction of Heart Failure (SENSE-HF) trial has previously demonstrated that intrathoracic impedance measurements have low sensitivity and positive predictive value (38.1%) for worsening heart failure status [19], and the Diagnostic Outcome Trial in Heart Failure (DOT-HF) showed that an audible patient alert in response to reduced intrathoracic impedance did not improve outcomes but was associated with more hospitalisations and urgent outpatients visits [20].

A limitation of these approaches is poor specificity. The risk of hospitalisation in the Program to Access and Review Trending Information and Evaluate Correlation to Symptoms in Patients with Heart Failure (PARTNERS-HF) was 5.5-fold higher if combined heart failure device diagnostic criteria were fulfilled (HR 5.5, 95% CI 3.4–8.8; *p* < 0.0001), but still only 4% within 30 days [21] Hence, despite considerable enthusiasm, especially during the coronavirus disease 2019 (COVID-19) pandemic where avoiding hospitalisations and outpatient visits became critical and led to an extensive roll-out of remote monitoring services, the data from trials suggest that increased activity may not consistently reduce admissions. These failings may be due to the frequency of data collection, the variables being collected, the specificity of the cut-points for intervention, or the efficacy of the interventions themselves. Nevertheless, risk profiling and patient management using remotely collected data from both external and implanted electronic devices continue to be a priority for patients and healthcare services around the world [22].

### Remote Haemodynamic Assessment

One potentially promising approach is remote haemodynamic assessment of pulmonary artery pressures by, for example, the CardioMEMs device (Abbott Laboratories, Illinois, USA). Although not originally designed to identify risk and avoid hospitalisation, rather to optimise the treatment of chronic heart failure, as we have suggested, these are one and the same aim. In the haemodynamic-GUIDEed management of Heart Failure (GUIDE-HF) trial, a responsive pulmonary artery pressure monitoring strategy was not associated with a lower rate of all-cause mortality or total heart failure events than usual care in patients at risk of hospitalisation (NYHA II-IV and hospitalisation within the previous 12 months or elevated natriuretic peptides) (hazard ratio 0.88, 95% CI 0.74–1.05; *p* = 0.16). Although the overall result of the trial was neutral, it was noted that the event rate in the active arm was lower during the COVID-19 pandemic than the period prior to this (*p*_interaction_ = 0.11). In a pre-specified analysis restricted to events prior to the pandemic, remote monitoring was associated with a reduction in the combined primary endpoint (HR 0.81, 95% CI 0.66–1.00; *p* = 0.049) [23].

## Questions Around Remote Monitoring

Two key questions in preventing the primary (or repeat) hospitalisation are whether the response can be delivered in a timely manner in the community, where its use achieves clinical stability swiftly enough and secondly, whether in a population of patients receiving disease-modifying therapies, an effective intervention exists which has not yet been employed. Even in GUIDE-HF with direct congestion monitoring, the overall results of the trial were neutral, possibly as it enrolled a well-treated and low risk population (cardiovascular mortality was 5% within 12 months of enrolment) of whom the majority did not have elevated pulmonary artery pressures at baseline and therefore had little scope to improve [24]. Despite the questions that remain, an admission with heart failure however classified could be used as a stimulus to consider device-based monitoring provided this is done with a focus on activities proven to reduce the risks of future events.

## How to Manage the ‘At Risk’ or Decompensated Patient

### Alleviation of Symptoms

Therapies for worsening heart failure should stabilise haemodynamics and relieve symptoms. For this reason, loop diuretics remain the cornerstone of management. These agents increase renal excretion of sodium and water, alleviating congestion, the most common symptom of worsening heart failure. In doing so, they can improve cardiac contractility by moving left ventricular haemodynamics into a more favourable position of the Frank Starling curve. For patients at risk of hospitalisation, doses of these medications can be increased by the oral route, or administered intravenously in the community, although for many, hospitalisation will be necessary or more practical. Although studies defining the optimal dosing, frequency, and whether bolus or infusions are preferred are limited, what limited evidence there is suggests that these should be tailored to the individual patient [25]. Clinical judgement is required, and it is advisable to start at lower doses, increasing where necessary.

Combination therapy can achieve additional diuresis with thiazide diuretics and mineralocorticoid receptor antagonists having been used alongside loop diuretics for decades. More recently, the ADVOR (Acetazolamide in Decompensated Heart Failure with Volume Overload) trial showed the addition of acetazolamide to intravenous loop diuretics resulted in more successful decongestion within 3 days (risk ratio 1.46, 95% CI 1.17–1.82; *p* < 0.001) and reduced length of hospital stay [26]. Acetazolamide is a carbonic anhydrase inhibitor which reduces proximal tubular sodium reabsorption, increasing natriuresis and diuresis beyond what can be achieved by loop diuretics alone [27]. Regardless of how diuresis is achieved, persistent congestion at discharge is a major predictor of death or rehospitalisation [28], and so sufficient diuresis prior to discharge and oral diuretics at doses to maintain clinical stability are advised.

Intravenous vasodilators alleviate congestion by dilating venous and arterial vessels, resulting in reductions in afterload [1]. Although a nitrate infusion was long considered standard of care, two recent trials have questioned the value of routine administration in which outcomes and symptoms were similar comparing early and sustained intravenous vasodilators to usual care with diuretics [29, 30].

### Supportive Care

Supplemental oxygen is not recommended routinely due to the risk of vasoconstriction and reduced cardiac output but is required for patients presenting with acute pulmonary oedema who have reduced peripheral oxygen saturations. Where supplementary oxygen in the ward setting is insufficient, continuous positive airway pressure (CPAP) or non-invasive ventilation (NIV) rapidly correct respiratory distress, hypercapnaenia, and acidosis, when used alongside intravenous diuretic therapy although these have not been shown to improve survival [31].

Patients presenting with cardiogenic shock require circulatory support using inotropes or vasopressors to maintain cardiac output, often used in combination. Inotropes increase cardiac contractility, thereby increasing cardiac output, whilst vasopressors increase peripheral vascular resistance, increasing end-organ perfusion but at the expense of increased afterload. Vasopressors, especially those with adrenergic mechanisms, can result in tachycardia, myocardial ischaemia, and arrythmia, and their use is associated with worse outcomes [32], whilst clinical trials are limited to heterogenous populations in which benefits were not shown [33, 34]. As a result, guidelines provide a class III recommendation in patients with systolic blood pressure ≥ 90 mmHg with these agents reserved for patients with left ventricular systolic dysfunction, low cardiac output, and hypotension [1]. Levosimendan is an alternative agent which has both inotropic and vasodilatory properties and results in improved cardiac output and reduced fillings pressures. Despite these favourable pharmacological proportions, levosimendan is not associated with improved survival compared to dobutamine in patients with ADHF [35], although repetitive infusions are used in some settings as a bridge-to-transplant in those dependent on dobutamine infusions [36].

## How to Modify Prognosis for the Hospitalised Patient

### Initiation of Disease Modifying Pharmacological Therapies

Therapies which improve acute haemodynamics or strategies to optimise the delivery of care for the decompensated patient have not translated into meaningful improvements in outcomes, suggesting that more completely treating the underlying syndrome should be our focus instead. For patients with heart failure with reduced ejection fraction (HFrEF), four classes of medications targeting the neurohormonal maladaptations of the syndrome are proven to reduce hospitalisations and improve survival [37]. In clinical practice, it typically takes many months before patients receive optimised doses of indicated pharmacological therapies, and many never do [38]. Hospitalisation with worsening heart failure offers the opportunity to initiate and rapidly optimise disease modifying pharmacological therapies, whilst also ensuring patients receive these agents during the period of highest risk.

Of particular relevance to hospitalised patients are sodium glucose co-transporter 2 inhibitors (SGLT2i) which are proven to improve symptoms, reduce hospitalisation, and extend longevity [39, 40]. A consistent result from clinical trials assessing these agents are the very early benefits, with differences in hospitalisations and deaths observed within the first 28 days. This has prompted calls for these agents to be given equal priority to more established therapies [41], with this approach reflected in recent guidelines [1]. Trials and observational cohort studies have shown that SGLT2i can be safely initiated during a hospitalisation with worsening heart failure and are associated with improved outcomes [42, 43].

In PARADIGM-HF (Prospective Comparison of Angiotensin Receptor-Neprilysin Inhibitor [ARNI] and Angiotensin-Converting-Enzymes Inhibitor [ACEi] to Determine Impact on Global Mortality), sacubitril-valsartan was shown to be superior to enalapril with respect to cardiovascular mortality and hospitalisations with heart failure (HR0.8, 95% CI 0.73–0.87) for ambulatory patients chronic heart failure and LVEF ≤ 40% (amended to ≤ 35% during the trial) [44] who had previously been able to tolerate treatment with an ACEi. Treatment with ARNI is currently recommended for patients who have persistently impaired LVEF and symptoms despite treatment with an ACEi or angiotensin receptor blocker [1]; however, there have been calls for ARNI to be given greater priority, particularly for hospitalised patients. Two studies have investigated the initiation of sacubitril-valsartan during or shortly after hospitalisation with heart failure, demonstrating this agent can be safely intiated [45] with greater reductions in natriuretic peptides compared to enalapril [46]. Many patients in these trials were new diagnoses and ACEi or angiotensin receptor blocker naïve. Hence, ARNI may be considered for patients with de novo HFrEF (class IIb recommendation) [1].

Whilst therapies have been mainly limited to patients with a reduced ejection fraction, there are now data showing reductions in hospitalisations for worsening heart failure for those with a preserved ejection fraction [47]. Additionally, post hoc analyses of the relevant trials suggest that although the efficacy of ARNI appears to be attenuated at higher LVEF, benefits extend to patients who would be considered to have mildly reduced ejection fraction [48], meaning therapies for those with LVEF > 40% are no longer limited to alleviation of symptoms and treatment of comorbidities.

### Cardiac Implantable Electronic Devices

Cardiac resynchronisation therapy (CRT) is amongst the most effective treatments for HFrEF. For indicated patients who despite optimised medical therapy have persistently impaired left ventricular ejection fraction (LVEF), ongoing symptoms, and QRS duration ≥ 130 ms [1], receipt of CRT is associated with a spectrum of improvement or stabilisation, reductions in heart failure hospitalisations, and mortality [49]. Implantation of CRT during a hospitalisation with worsening heart failure has the potential to alter the subsequent clinical course, whilst providing an effective treatment with immediate haemodynamic benefits during the post-discharge vulnerable phase.

Implantable cardioverter defibrillators (ICD) reduce the risk of sudden cardiac death and all-cause mortality for patients fulfilling similar criteria (with or without QRS ≥ 130 ms) [50]. Although patients may be particularly vulnerable following discharge, these devices should be reserved for those with established HFrEF, who have not remodelled despite optimal pharmacological therapy. For patients presenting with new onset HFrEF (which is associated with a more favourable prognosis) or for those whose medical therapy has not been optimised, providing protection during this vulnerable phase must be balanced against the risks of unnecessary implantation for patients who subsequently improve. All devices introduce the risk of device-related complications, with ICD-specific complications including inappropriate shocks, shorter battery longevity, and greater risk of lead failure.

A possible compromise, especially for patients with new HFrEF as a consequence of myocardial infarction, wearable cardioverter-defibrillators may be considered although these have not been proven to reduce the risk of morality and carry a risk of inappropriate shocks [51]. On the other hand, for those patients with an indication for CRT who are at high-risk of sudden cardiac death, and might otherwise be considered for an ICD, deferring treatment may be disadvantageous, especially considering that a broad QRS (especially left bundle branch block) is a consistent predictor of worse outcomes and failure to remodel in response to medical therapy [52], and so inpatient implantation of a CRT-defibrillator might be considered reasonable in selected patients.

### Structural Interventions

For patients hospitalised with worsening heart failure in whom the underlying pathophysiology is a primary valvular disorder, standard risk assessment and surgical procedures should be considered. However, many hospitalised patients, particularly those who have left ventricular systolic dysfunction are unlikely to be considered suitable for surgical treatments. Of particular relevance, severe aortic stenosis has a particularly poor prognosis in the presence of the heart failure syndrome or left ventricular systolic dysfunction. Non-surgical options for the management of aortic stenosis include balloon valvuloplasty and transcatheter aortic valve implantation (TAVI), with retrospective analyses suggesting the risk of re-hospitalisation and subsequent interventions is lower in those undergoing immediate rather than delayed TAVI, even after adjustment for relevant confounders [53].

Many patients with HFrEF develop secondary mitral regurgitation, in which annular dilatation due to ventricular or atrial distension results in the failure of morphologically normal valve leaflets to coapt [54]. Patients with HFrEF and any degree of mitral regurgitation have greater symptoms, higher rates of hospitalisation, and worse survival than those without [55, 56]. However, on average, they have more impaired left ventricular function, and whilst pharmacological and device therapies targeting the underlying pathophysiology reduce secondary mitral regurgitation [49], whether treating targeting secondary mitral regurgitation improve outcomes is unclear. Trials of edge-to-edge repair of secondary mitral regurgitation using the Mitra-clip (Abbott Laboratories, Illinois, USA) device reached divergent results [57, 58] which may be explained by the differing characteristics of patients enrolled in these trials, or the plausible but unproven concept of proportionate and disproportionate mitral regurgitation [59]. Percutaneous treatment of secondary mitral regurgitation may be considered in selected patients who remain symptomatic despite optimised pharmacological and device therapies and who are not eligible for surgical repair or replacement to improve symptoms and reduce the risk of future hospitalisation [60].

## Reassessing Goals of Care

**Fig. 3:**
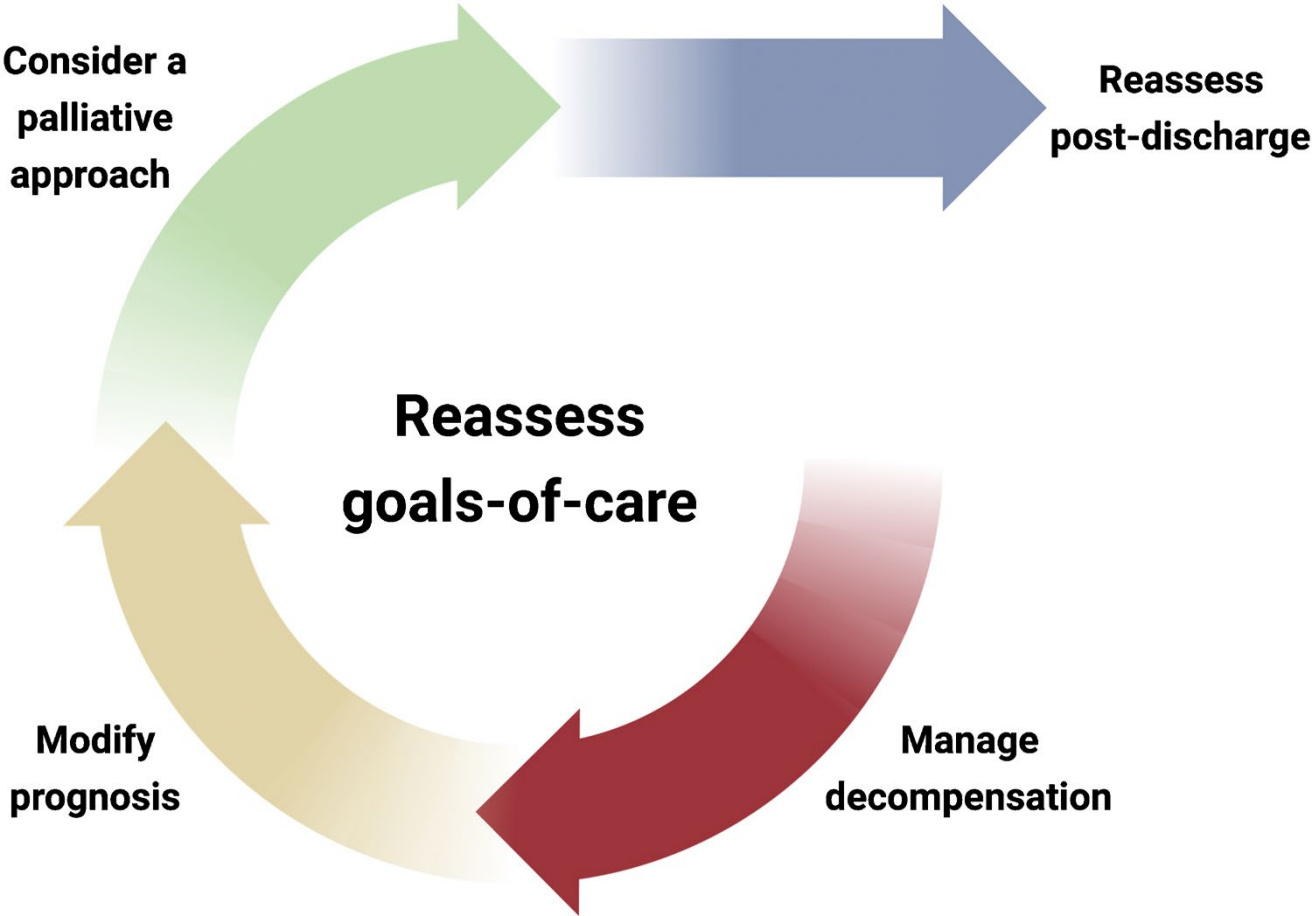
Ongoing reassessment of goals-of-care. Hospitalisation should be viewed as an opportunity to continually reassess the goals-of-care. Management of decompensation, optimisation of prognostic therapies, and the concurrent palliative approach should be considered

Heart failure is a chronic disease which cannot be cured [2], and so even if we were to give greater priority to optimising pharmacological and devices therapies, we must accept that the risk of subsequent re-hospitalisation and mortality is likely to be reduced but not eliminated. For many patients, particularly those who are elderly, frail, or have other life-limiting comorbidities, the benefits of pharmacological and device therapies come with a greater risk, and a greater benefit to patients’ quality of life might be achieved by an early integration of a palliative approach [61]. Such an approach focuses on the management of symptoms, as well as facilitating advanced care planning, taking into account patient preferences on preferred place of care and the appropriateness of readmission to hospital [1]. Re-evaluating goals-of-care daily during an admission crucial given disease trajectories can change rapidly during episodes of worsening heart failure (Fig. 3).

Various methods have been proposed to help clinicians identify patients who might benefit from such an approach. One such prompt is the ‘surprise question’ — “would you be surprised if this person were to die within the next 12 months?”. This question has been validated in a number of chronic diseases, including patients hospitalised with heart failure for whom it reliably identifies nearly all of those likely to die (sensitivity 85%), whilst also accuracy identifying who is unlikely to die (negative predictive value 88%) and can be used with similar levels of accuracy by a range of healthcare professionals in both inpatient and outpatient settings [62, 63]. Whilst the surprise question is more accurate than the New York Heart Association classification and avoids the drawbacks of more complex tools, by asking whether death is possible (rather than probable), there is a risk that patients may be incorrectly classified. However, this is unlikely to be detrimental to outcomes where the question is used as a prompt to consider advanced care planning and palliative care interventions since these are generally applied concurrently with usual care [64].

## Optimising Care During the Post-discharge ‘Vulnerable’ Phase

Many patients discharged from hospital following an episode of worsening heart failure are not receiving all indicated classes of guideline-directed medical therapy [65]. Even where these are prescribed, they are rarely done so at evidence-based doses [66]. The approach taken by many has been to focus care on the alleviation of congestion during hospitalisation, whilst deferring the initiation and optimisation of pharmacological therapies until after discharge. Evidence from registry studies suggests that even in high-income countries, if patients leave hospital not receiving these agents new classes of medications are rarely initiated and doses seldom increased during the post-discharge ‘vulnerable phase’ [66]. On the other hand, once prescribed, these agents are usually not discontinued, with subsequent up-titration associated with better outcomes [38]. Heart failure nurse specialists, in particular, have a key role in the management of patients transitioning from hospital based care to the community. Intensified follow-up with re-evaluation of symptoms, fluid status, and disease trajectory are essential, as well subsequent dose optimisation and reassessment of cardiac structure and function to plan future interventions and guide prognosis.

## Conclusions

Worsening heart failure events identify patients as being high risk for subsequent poor quality of life, re-hospitalisation, and mortality. Much of the adverse prognosis is related to the severity of underlying disease, rather than decompensation events themselves. Moving forwards, efforts to optimise disease modifying pharmacological therapies, devices, and structural interventions during decompensation episodes, have the potential to further improve outcomes for patients at the greatest risk. Those caring for those with chronic heart failure should be cognisant that for many, prognosis may be irreversible and the adoption of an early, palliative approach alongside active care may be most effective means by which to improve quality of life.
